# Does damage to hypothalamic paraventricular nucleus underlie symptoms of ultradian rhythm disorder and an increased anxiety in coronavirus disease 2019?

**DOI:** 10.3325/cmj.2020.61.377

**Published:** 2020-08

**Authors:** Ivana Rosenzweig, Dinko Mitrečić, Zdravko Petanjek, Bobby Duffy, Allan H. Young, Alexander D. Nesbitt, Mary J. Morrell

**Affiliations:** 1 Sleep and Brain Plasticity Centre, Department of Neuroimaging, Institute of Psychiatry, Psychology and Neuroscience (IoPPN), King's College London, London, UK; 2 Sleep Disorders Centre, Guy's and St Thomas’ NHS Foundation Trust, London, UK *ivana.1.rosenzweig@kcl.ac.uk*; 3 Laboratory for Stem Cells, Croatian Institute for Brain Research, University of Zagreb School of Medicine, Zagreb, Croatia; 4 Center of Excellence for Basic, Clinical and Translational Neuroscience and Department of Anatomy and Clinical Anatomy, University of Zagreb School of Medicine, Zagreb, Croatia; 5 The Policy Institute, King's College London, London, UK; 6 Department of Psychological Medicine, Institute of Psychiatry, Psychology, and Neuroscience, King's College London, London, UK; 7 The National Heart and Lung Institute, Imperial College London, London, UK

Long-term physical and psychological effects of the coronavirus disease-2019 (COVID-19) are yet to be fully understood, and some of them could be due to direct central nervous system involvement. Severe acute respiratory syndrome coronavirus-2 (SARS-CoV-2) may directly target parts of the brain, more specifically the hypothalamus and its paraventricular nucleus, and possibly lead to increased prevalence of anxiety disorders.

There is increasing recognition that the COVID-2019 may, in some vulnerable individuals, induce prolonged post-viral fatigue states, characterized by the aberrant daytime oscillation in alertness, disturbed sleep cycles, and significant fluctuating anxiety. We propose that the striking ultradian nature of these fatigue states could be due to SARS-CoV-2 direct effect on the hypothalamic paraventricular nucleus. We also propose that dissemination of SARS-CoV-2 to the hypothalamus may occur via brain’s sub-fornical organ during any phase of the infection, and that this direct monosynaptic route may lead to SARS-CoV-2’s binding to the angiotensin-converting-enzyme-2-receptor-tagged part of the paraventricular nucleus. Any ensuing localized neurotoxicity in the pivotal region of the paraventricular neurocircuitry may cause significant imbalance in its key stress, arousal, and metabolic outputs, with important consequences to our body’s physiological responses to psychological and physical stress.

There has been increased media coverage and reports of significant COVID-19 post-viral fatigue, aberrant daytime oscillation in alertness, and (often irresistible) waves of episodic anergia (1). There are reports of subjective symptoms that continue for weeks, sometimes months, and that include episodic headaches and increased fluctuating daytime anxiety, combined with disturbed nocturnal sleep cycles, punctuated by discernible panic attacks (eg, pavor nocturnus), leading to hypersomnia, or insomnia, in affected patients (1-3). This clinical picture appears to persist in an episodic, if milder, manner, even once the main symptoms of the SARS-CoV-2 infection have long subsided. Anecdotally, non-hospitalized patients with mild to moderate COVID-19 similarly report prominent disruption of the 1–2 hours ultradian basic rest-activity cycle (BRAC) (4). The striking ultradian nature of this clinical picture is, as of yet, unclear.

Ultradian rhythms have long been demonstrated in various electroencephalographic frequencies, in the tendency to fall asleep during the day, and in autonomic indices of arousal, including respiratory and heart rates (4). Moreover, mood and cognitive fluctuations in several major neurologic (eg, Alzheimer disease) and psychiatric disorders (eg, depression), cluster headaches, rapid-eye-movement (REM)–non-REM sleep cycles, hypothalamic-pituitary-axis hormone release, nasal cycles, locomotion, and body temperature have all been suggested to follow ultradian cycles with similar periodicities (4).

**Figure 1:**
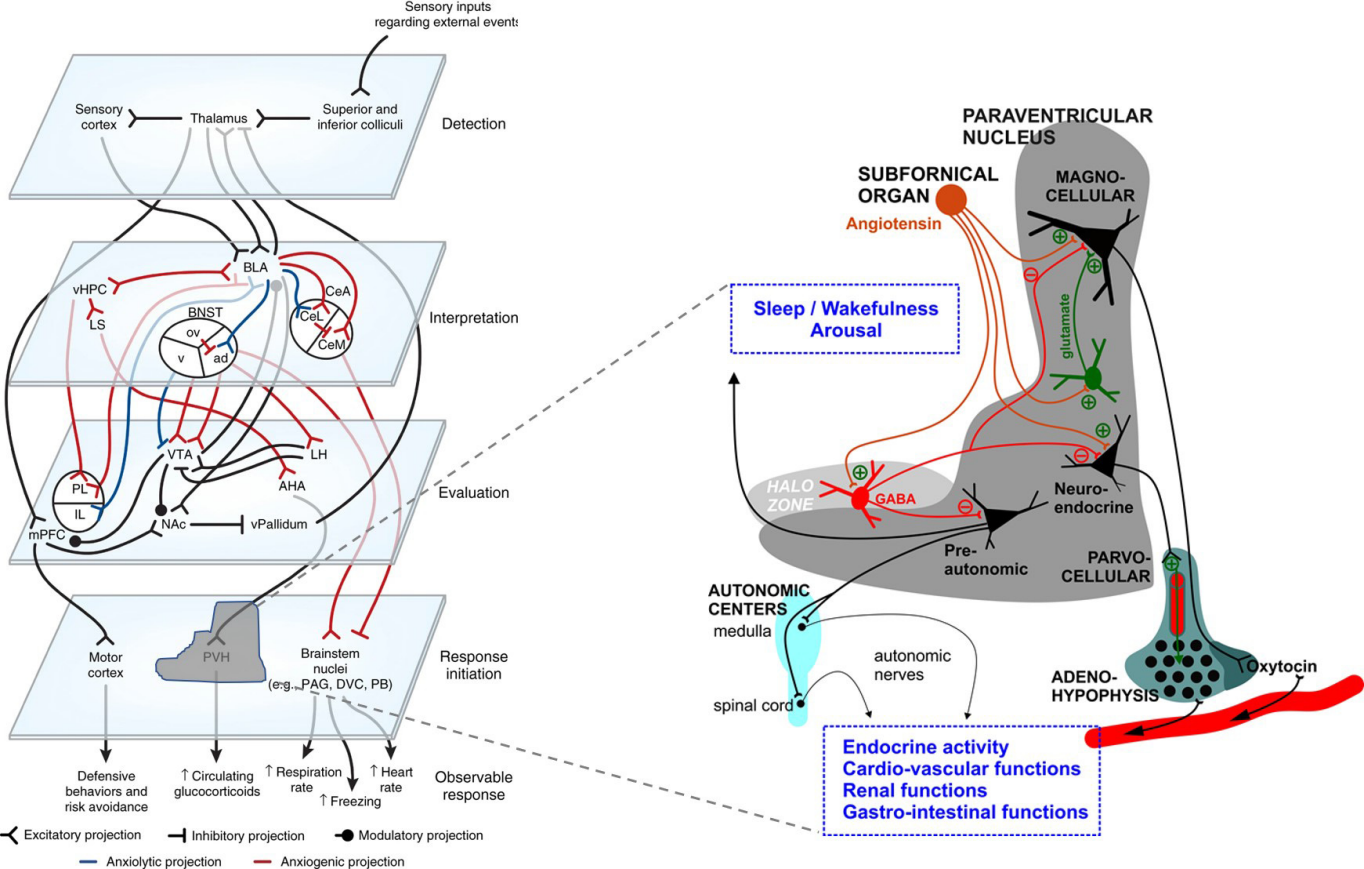
The schematic illustration of the basic neuronal organization of the paraventricular nucleus (PVN) of the hypothalamus depicting its purported role in a four-step model of neurocircuitry of anxiety. On the left, a four-step model by Calhoon and Tye (7) is shown: according to this model external events are detected, interpreted, evaluated, and responded to by succeeding levels of highly interconnected neural circuits (adapted with permission from 7). Events are further interpreted as threatening or nonthreatening depending on the balance between opposing circuits; when the balance is shifted toward projections interpreting events as threatening, this leads to anxiety. On the right, PVN’s neurocircuitry is depicted. Afferent inputs to the nucleus arrive from many important integrative centers of the medulla, pons, and hypothalamus. The subfornical organ is among major inputs with identified angiotensin release acting directly on magnocellular and parvocellular neurons, or indirectly via intranuclear circuitry that includes inhibitory gamma-aminobutyric-acid (GABA) (red) and excitatory glutamate (green) interneurons. Changes within paraventricular angiotensin subcircuitries are described under stress condition and are reflected on release of stress hormone and arousal. GABA interneurons have been shown to express angiotensin-converting-enzyme-2 (ACE2) (11), and as such might be targeted by severe-acute-respiratory-syndrome-coronavirus-2 (SARS-CoV-2). The majority of GABA interneurons have been localized to the halo zone surrounding the PVN, and their role as an additional gatekeeper and integrator in controlling the excitability of PVN outputs has been proposed (8). Any changes in the PVN circuitries, due to their major control over most of neuro-endocrine axes and neuronal autonomic centers, may cause robust alteration in homeostatic regulation, and through influence on regulatory brain centers impact on sleep and wakefulness, increased propensity to affective disorders and anxiety, and alteration of ultradian rhythms. Abbreviations: ad – anterodorsal nucleus of the BNST; ACE2 – angiotensin-converting-enzyme-2; AHA – anterior hypothalamic area; BLA – basolateral amygdala; BNST – bed nucleus of the stria terminalis; CeA – central amygdala; CeL – lateral subdivision of the central amygdala; CeM – centromedial subdivision of the amygdala; DVC – dorsal vagal complex; GABA – gamma-aminobutyric-acid; IL – infralimbic division of the mPFC; LH – lateral hypothalamus; LS – lateral septum; mPFC – medial prefrontal cortex; NAc – nucleus accumbens; ov – oval nucleus of the BNST; PAG – periaqueductal gray; PB – parabrachial nucleus; PL – prelimbic division of the mPFC; PVH/PVN – paraventricular nucleus of the hypothalamus; SARS-CoV-2 – severe-acute-respiratory-syndrome-coronavirus-2; v – ventral BNST; vHPC – ventral hippocampus; vPallidum – ventral pallidum; VTA – ventral tegmental area.

A recent study by King’s College London (KCL) has highlighted a significant impact of the current COVID-19 pandemic on the quality of sleep and anxiety levels in the United Kingdom (UK) (5). Substantial changes to sleep and anxiety patterns have been reported since the UK government announced lockdown measures. Findings from a survey of 2254 UK residents, aged 16-75, suggest that six in ten people have suffered from disturbed, unrefreshing sleep, of both shorter and longer duration, with more vivid dreams (5). The findings of the KCL survey and patients’ reports are perhaps unsurprising, given the unprecedented situation and enormous adjustments we are undergoing as a society. However, we argue that SARS-CoV-2 might also directly contribute to described disturbed ultradian patterns and increased anxiety through its binding to neuronal angiotensin-converting-enzyme-2 (ACE2)-receptors. SARS-CoV-2 can directly decrease ACE2 activity and lead to cell toxicity in other tissues. This interaction is believed to subsequently lead to a myriad of symptoms, from increased blood pressure to kidney failure (3,6). We correspondingly argue a direct SARS-CoV-2 effect on the specific part of the brain’s hypothalamic paraventricular nucleus (PVN) circuitry (Figure 1) as a neurologic mechanism that may, at least in part, underlie previously reported ultradian disruption, and that may lead to an increased anxiety symptomatology (7).

It is now widely accepted that COVID-19 pathology may include CNS involvement (6). Dissemination of SARS-CoV-2 across the cribriform plate of the ethmoid bone, or via some other CNS route (eg, area postrema, subfornical organ), may occur during any phase of the infection (2,6). Subsequently, cells of the nervous system, including those controlling BRAC-ultradian-rhythms might be damaged by impairment of the blood-brain-barrier, by the indirect damage brought by hypoxia/cerebral edema and intracranial hypertension, or indeed by direct SARS-CoV-2-neurocytotoxic effects. We propose that SARS-CoV-2 may target the distinct ACE2-tagged part of the PVN-subcircuitry (8), via a direct monosynaptic subfornical route (Figure 1), leading to the pleomorphic dysautonomic ultradian presentation, which is further compounded with nocturnal sleep fragmentation.

The PVN is an important ultradian master oscillator that has been proposed to regulate BRAC and the body’s physiological responses to psychological and physical stress by initiation of a hormone cascade that eventually alters physiological and metabolic states (9,10). Its role in modulation and triggering of the trigeminal autonomic cephalalgias by mediating the regulation of nociceptive and autonomic input, has similarly been proposed. Intriguingly, several recent studies have suggested a more versatile role for PVN neurons in acute behavioral changes after stress, by encoding the valence of encountered stimuli (7,9). Neuronal ACE2 receptors, the presumed SARS-CoV-2 target via monosynaptic subfornical connections, are specifically expressed on a distinct subpopulation of GABA-ergic-interneurons within PVN’s circuitry (11). The GABA-interneurons act as the supernumerary (fine-tuning) gatekeepers that ensure a pivotal integration of the excitability of all PVN’s major autonomic outputs (8). PVN’s outputs (Figure 1) include those that control key stress, arousal, and metabolic functions (7).

In conclusion, given the role of PVN as a major neurocomputational hub that ensures adequate signal to noise integration and timely processing of multiple physiologic signals, any SARS-CoV-2-ACE2 interaction within this circuitry may have significant consequence. It can significantly affect short-term biological rhythms that are crucial in regulating preparedness of our physiological functions for external world’s unpredictability. For example, in affected patients this might manifest itself with increased physiological and emotional fragility, dysautonomia, and more specifically, with an increased anxiety (7). Anxiety is an important aspect of the emotional repertoire that aids awareness and enables rapid responses to possible hazards (7). However, when physiological changes including sweating, dizziness, and increases in blood pressure and heart rate (7) are triggered by stimuli that do not pose immediate danger, or that are internally inappropriately generated, this can be highly debilitating (7). Future multimodal imaging clinical and preclinical studies are needed to validate our hypothesis, to further elucidate this specific ultradian post-viral phenotype and the idiosyncratic individual sensitivity, as well as to help toward the long-term rehabilitation of the affected patients.
